# Eating Disorders, Physical Fitness and Sport Performance: A Systematic Review

**DOI:** 10.3390/nu5125140

**Published:** 2013-12-13

**Authors:** Marwan El Ghoch, Fabio Soave, Simona Calugi, Riccardo Dalle Grave

**Affiliations:** Department of Eating and Weight Disorders, Villa Garda Hospital, Via Montebaldo, Garda (VR) 89I-37016, Italy; E-Mails: fabio.soave@yahoo.it (F.S.); si.calugi@gmail.com (S.C.); rdalleg@tin.it (R.D.G.)

**Keywords:** anorexia nervosa, bulimia nervosa, eating disorders, muscle function, physical fitness, sport performance, functional capacity, VO_2_max, athletic performance, muscle strength, physical exercise

## Abstract

Background: Eating disorders are health problems that are particularly prevalent in adolescents and young adults. They are associated with considerable physical health and psychosocial morbidity, and increased risk of mortality. We set out to conduct a systematic review to determine their effect on physical fitness in the general population and on sport performance in athletes. Methods/Design: A systematic review of the relevant peer-reviewed literature was performed. For inclusion, articles retrieved from PubMed had to be published in English between 1977 and 2013. Wherever possible, methods and reporting adhere to the guidelines outlined in the PRISMA statement. Some additional studies were retrieved from among those cited in the reference lists of included studies and from non-electronic databases. Literature searches, study selection, method and quality appraisal were performed independently by two authors, and data was synthesized using a narrative approach. Results: Of the 1183 articles retrieved, twenty-nine studies met the inclusion criteria and were consequently analysed. The available data indicate that eating disorders have a negative effect on physical fitness and sport performance by causing low energy availability, excessive loss of fat and lean mass, dehydration, and electrolyte disturbance. Discussion: Although the paucity of the available data mean that findings to date should be interpreted with caution, the information collated in this review has several practical implications. First, eating disorders have a negative effect on both physical fitness and sport performance. Second athletics coaches should be targeted for education about the risk factors of eating disorders, as deterioration in sport performance in athletes, particularly if they are underweight or show other signs of an eating disorder, may indicate the need for medical intervention. However, future studies are needed, especially to assess the direct effect of eating disorders on sport performance.

## 1. Introduction

In the general population, eating disorders have a lifetime prevalence of roughly 0.6% for anorexia nervosa, 1% for bulimia nervosa, and 3% for binge eating disorder [1,2,3]. Little is known about the distribution of eating disorder not-otherwise-specified (NOS) in the community, although 40% to 70% of eating disorder patients seeking treatment are diagnosed as having this condition [4,5]. Eating disorders are particularly common in adolescents and young adults, and also seem to be more prevalent among athletes than in general population. Indeed, the latest, largest and best-designed study on top athletes found an overall prevalence of eating disorders of 13.5%, far higher than figures reported for the general population. This study found that the prevalence of eating disorders in athletes was considerably higher in females (20.1%) than in males (7.7%) [6]. Similarly high percentages were documented in a British study on female distance runners, in which 29 out of 184 female athletes (16%) were diagnosed with an eating disorder of clinical severity. Of these, 3.8% had anorexia nervosa, 1.1% bulimia nervosa and 10% eating disorder NOS [7]. The fact that many individuals with eating disorders do not seek professional help, as observed in community studies conducted in the USA [3], complicates epidemiological research on the issue.

Eating disorders carry an increased risk of death [8,9], and are often associated with significant physical health complications [10]. These complications arise through three main mechanisms, which often operate in concert: undereating, purging, and low body weight [11]. In athletes, disordered eating, low weight, and low energy intake have been associated with a condition described as “female athlete triad syndrome”, which presents as a cluster of three clinical features: menstrual dysfunction, low energy availability, and reduced bone mineral density [12]. The prevalence of all three features of this syndrome in athletes is about 4.3%, not greatly different from that observed in healthy controls (3.4%) [13], but the presence of at least two components out of three is seen in 5.4% to 26.4% [13].

As well as physical manifestations, eating disorders also have a negative effect on psychosocial functioning. For example, the overvaluation of shape, weight, and eating control (*i.e*., self-assessment based predominantly or exclusively on these factors) and its expressions, considered the core psychopathology of eating disorders [14] has a negative effect on the ability to be around others and have intimate relationships (interpersonal functioning). Extreme concern about eating control and its expressions also negatively affects mood and cognition [14], in severe cases damaging education and vocational performance [1], with up to 20% being no longer able to function independently 10–20 years after the onset of the illness [15]. Such secondary physical and psychosocial effects can be extremely disabling, and are also likely to negatively affect physical fitness in individuals with eating disorders and sport performance in professional athletes. Despite the importance of the issue, this topic has not yet been addressed in any great depth.

## 2. Aim and Objectives of the Systematic Review

This review is designed to systematically interrogate the published literature to address the clinical issues of whether eating disorders compromise physical fitness in non-athletes, and whether eating disorder features negatively affect sport performance in athletes, according to the PICO formulation [16].

P-Population: individuals in the general population and athletes affected by eating disorders or having eating disorders features. I-Intervention: (i) weight restoration or recovery from eating disorders; (ii) physical activity programme with or without weight restoration. C-Comparison: eating disorder group before and after weight restoration or recovery (when data is available), and healthy control group (when available). O-Outcome: physical fitness and sport performance parameters.

## 3. Methods/Design

This report was completed in accordance with the Preferred Reporting Items for Systematic Review and Meta-Analyses (PRISMA) guidelines [17].

### 3.1. Inclusion and Exclusion Criteria

All studies that evaluated the effect of either eating disorders or eating disorder features on physical fitness and sport performance were taken into consideration. However, the review included only: (i) manuscripts in English; (ii) original articles; (iii) prospective or retrospective observational (analytical or descriptive), experimental or quasi-experimental studies. Non-original studies, including editorials and letters to the editor, were excluded.

### 3.2. Information Source

Literature searches were initially performed using PubMed only, but, given the paucity of papers that met the selection criteria, especially those on the relationship between sport performance and eating disorders, the search was extended to books and non-electronic databases.

### 3.3. Search Strategy

The following MeSH terms, words and combinations of words, were used in constructing the systematic search: #1 eating disorders, #2 anorexia nervosa, #3 bulimia nervosa, #4 physical fitness, 5# muscle function, #6 functional capacity, #7 VO_2_, #8 sport performance, #9 athletic performance, #10 muscle strength, #11 physical exercise, as well as their intersections: (#1 OR #2 OR #3) AND (#4 OR #5 OR #6 OR #7 OR #8 OR #9 OR #10 OR #11). Additionally, reference lists of key studies were searched manually to retrieve any that had not been identified via the initial search strategy. The search yielded articles published between 1977 and 2013.

### 3.4. Study Selection

Electronic and non-electronic literature searches, study selection, methodology and appropriateness for inclusion were performed independently and in duplicate by two authors (E.M. and C.S.). Quality appraisal was conducted according to the NICE guidelines checklist [18]. In references derived from books and non-electronic databases quality was not assessed, as it was not possible to apply the NICE guidelines checklist or another quality appraisal tool. In these cases, the publications have still been included because they were deemed relevant to the topic and met the inclusion criteria discussed previously. Disagreements between reviewers were resolved by consensus.

### 3.5. Data Collection Process and Data Items

As a first step, the title and abstract of each paper were screened for language and relevance of subject matter. In the next round, studies were assessed for methodological quality and appropriateness for inclusion. Details of the selected studies are presented in Table 1 and Table 2, which report the authors, year of publication, type, sample and main findings of each study.

### 3.6. Data Synthesis

Studies deemed fit for inclusion were subjected to narrative synthesis. A narrative review is discursive in nature and seeks to summarize the current state of knowledge in relation to a particular domain by considering a wide variety of sources and reaching conclusions through reason or argument [19].

## 4. Results

The initial search retrieved 1183 papers. After analysing their titles and abstracts, 1159 papers were excluded because: 96 were not in English; 733 were not concerned with eating disorders, physical fitness and sport performance; 225 dealt with eating disorders or eating disorder features in the general population and athletes but made no reference to physical fitness or sport performance; 105 concerned eating disorders and performance but not athletic or sport performance (*i.e*., cognitive, psychological, memory, intellectual, and/or executive functioning and performance).

**Figure 1:**
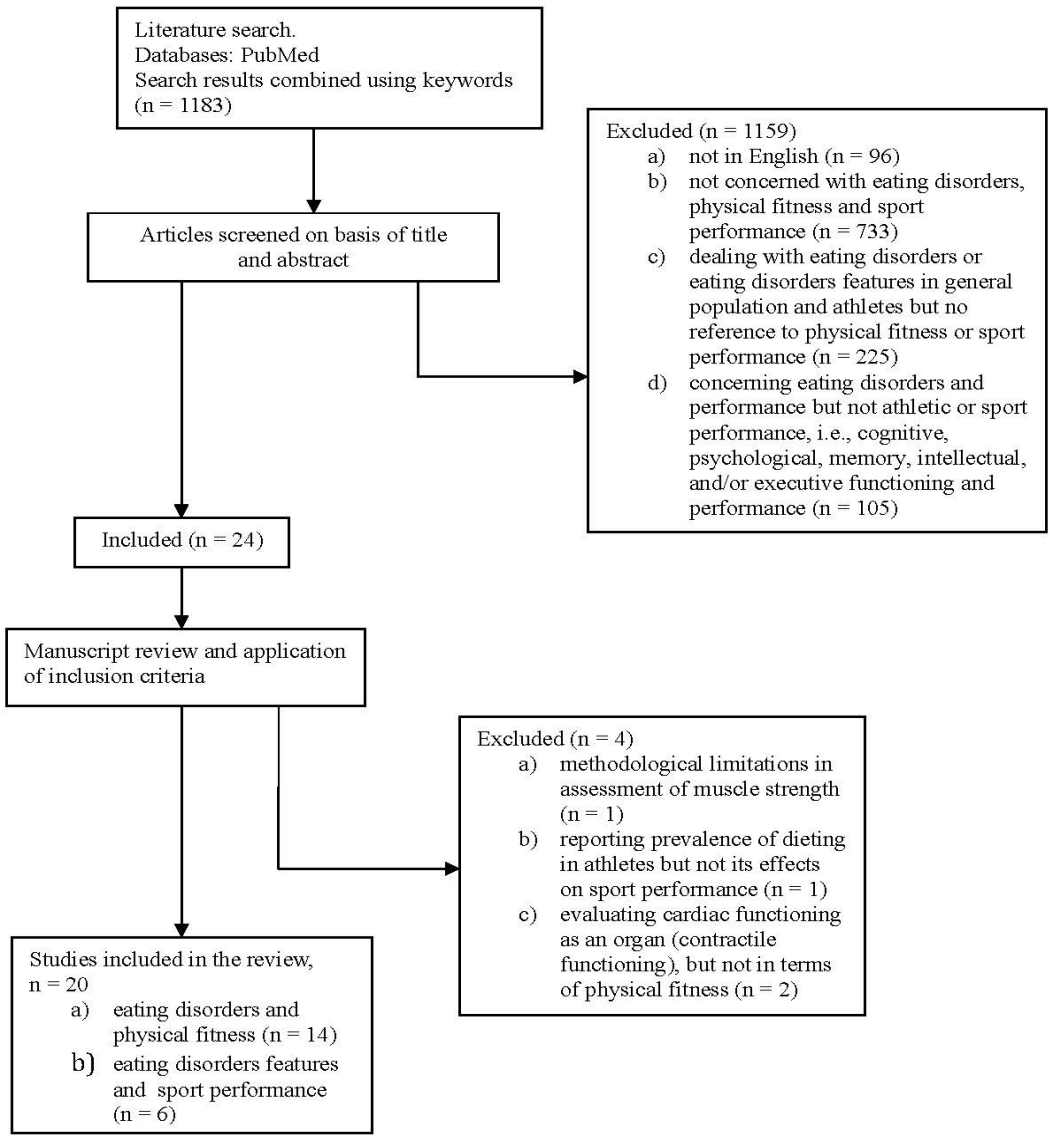
Flow chart summarizing the study selection procedure; An additional 9 studies included in the review were found in books and non-electronic databases.

Twenty-four articles remained, and were screened in light of their methodological quality and findings. At this stage four studies were excluded because: there were methodological limitations in their assessment of muscle strength (*n* = 1); they reported the prevalence of dieting in athletes but not its effects on sport performance (*n* = 1); and they evaluated the functioning of the heart as an organ (contractile functioning), but not in terms of physical fitness (*n* = 2) (Figure 1). Nine additional references on the relationship between sport performance and eating disorders were retrieved from books and non-electronic databases. Thus, in total, 29 references were retained for inclusion in the main body of the review.

The NICE guidelines checklist proved a fair to good quality for the studies that assessed physical fitness in eating disorders (*n* = 14) (mean score 5.64 points) (Table 3), and poor to fair quality for the studies that assessed the effects of eating disorders features on sport performance (*n* = 6) (mean score 3.83 points) (Table 4).

**Table 1:** Studies assessing physical fitness in eating disorders.

First Author	Year	Study	Sample	Main Results
Fohlin *et al*. [20]	1978	Cross-sectional	28 AN adolescents (17 F, 11 M)	Lower aerobic fitness than predicted values
Fohlin *et al*. [21]	1978	Longitudinal	8 AN adolescents (5 F, 3 M)	Pre-treatment reduction in aerobic capacity that is totally normalized with normal weight restoration
Nudel *et al*. [22]	1984	Cross-sectional	20 female AN adolescents and 15 controls	Reduced working capacity and cardiovascular response to exercise
Einerson *et al*. [23]	1988	Cross-sectional	42 AN females; 33 BN females and 14 lean controls	Lower muscle strength in AN than in BN and control groups Lower aerobic fitness in both AN and BN in comparison with control group
Waller *et al*. [24]	1996	Longitudinal	10 female AN inpatients	Partial improvement in aerobic capacity in eight weeks of re-feeding, but this remains lower than normal
Rigaud *et al*. [25]	1997	Longitudinal	15 AN patients (13 F, 2M) and 15 controls (13 F, 2 M)	Reduced muscle fitness (performance) in malnourished patients, which completely normalized after 45 days of re-feeding, although VO_2_ remained lower than in controls
McLoughlin *et al*. [26]	1998	Cross-sectional	8 AN females	Lower muscle strength than predicted values
Biadi *et al*. [27]	2001	Cross-sectional	19 AN females and 20 lean controls	Lower working capacity, cardiovascular response to exercise, and VO_2_ (aerobic fitness) at rest and during exercise in AN group than in control group
Rowland *et al*. [28]	2003	Cross-sectional	8 adolescent female AN inpatients and 8 controls	Low heart rate and lower aerobic fitness in AN patients than in controls
Chantler *et al*. [29]	2006	Longitudinal	14 female inpatient AN (7 trained, 7 not trained) and 7 trained controls	Increased muscular strength in AN patients who underwent an eight week light-intensity resistance training programme than in non-trained AN group
Fontana *et al*. [30]	2009	Cross-sectional	15 female AN inpatients, 15 female BN inpatients and 11 controls	No significant differences in postural stability between AN and control group BN group more unstable than control and AN groups
Bratland-Sanda *et al*. [31]	2010	Cross-sectional	59 longstanding female ED inpatients and 53 controls	Lower in muscular strength among longstanding ED (AN, BN and EDNOS) patients than in controls
Del Valle *et al*. [32]	2010	Longitudinal	22 AN adolescents (20 F, 2 M) divided into two groups (trained and not trained)	No significant improvement in functional capacity (including aerobic capacity) after 3-month low-moderate intensity resistance training programme
Alberti *et al*. [33]	2013	Longitudinal	37 female AN inpatients (adolescents and young adults) and 57 controls	Reduced physical fitness (aerobic, musculoskeletal, flexibility and motor) before weight restoration. Re-feeding and weight restoration improved physical fitness (but not muscular endurance), but this was still lower than in controls

Note: ED = eating disorder; AN = anorexia nervosa; BN = bulimia nervosa; EDNOS = eating disorder not otherwise specified; CT = control group.

**Table 2:** Studies assessing the effect of eating disorders features on sport performance.

Eating disorder feature	Studies (*n*)	First Author and Year	Results
Underweight	5	Boileau *et al*. [34] Cureton *et al*. [35] Clark *et al*. [36] Sherman *et al*. [37] Bonogofski *et al*. [38]	Conflicting and inconclusive results. Early studies reported that leaner individuals perform better, but this finding has not been confirmed in subsequent studies
Excessive and compulsive exercising	2	Ragalin *et al*. [39] Armstrong *et al*. [40]	Overtraining is frequent in athletes and may negatively influence sport performance
Short term dietary restriction	3	Ingjer *et al*. [41] Johnson *et al*. [42] Fogelholm *et al*. [43]	Transitory improvement of sport performance due to early starvation effects, with increased cortisol, adrenaline, noradrenaline, and VO_2_
Long term dietary restriction	1	Beals *et al*. [44]	Deterioration of sport performance due to glycogen depletion, increase in circulatory lactate, dehydration and loss of lean mass
Binge eating	1	Rankin *et al*. [45]	Inconclusive results, but binge eating seems to negatively influence sport performance if associated with excessive weight gain
Purging	3	Eichner *et al*. [46] Otis *et al*. [47] Thompson *et al*. [48]	Negative effect on sport performance through negative caloric balance, dehydration and hypokalaemia

**Table 3:** Evaluation of methodological qualities of studies assessing physical fitness in eating disorders.

Author *	Fohlin [20]	Fohlin [21]	Nudel [22]	Einerson [23]	Waller [24]	Riguad [25]	McLoughlin [26]	Biadi [27]	Rowland [28]	Chantler [29]	Fontana [30]	Bratland-S [31]	Del Valle [32]	Alberti [33]
Case series collected in more than one centre, i.e., multi-centre study	0	0	0	0	0	0	0	0	0	0	0	0	0	0
Is the hypothesis/aim/objective of the study clearly described?	1	1	1	1	1	1	1	1	1	1	1	1	1	1
Are the inclusion and exclusion criteria (case definition) clearly reported?	1	1	1	1	1	1	1	1	1	1	1	1	1	1
Is there a clear definition of the outcomes reported?	1	1	1	1	1	1	1	1	1	1	1	1	1	1
Were data collected prospectively?	0	0	0	0	1	1	0	1	0	0	1	0	0	1
Is there an explicit statement that patients were recruited consecutively?	0	0	0	0	1	1	0	1	0	1	1	1	1	1
Are the main findings of the study clearly described?	1	1	1	1	1	1	1	1	1	1	1	1	1	1
Are outcomes stratified? (e.g., by disease stage, abnormal test results, patient characteristics)	0	0	1	0	1	1	1	1	0	1	1	1	1	1
Total Score	4	4	5	4	7	7	5	7	4	6	7	6	6	7

Yes = 1, No (not reported, not available) = 0; Total score, 8; ≤3, poor quality; 4–6, fair quality; ≥7, good quality; * Named by reference number and listed in chronological order.

**Table 4:** Evaluation of methodological qualities of studies assessing the effect of eating disorders features on sport performance.

Author *	Boileau [34]	Cureton [35]	Clark [36]	Armstrong [40]	Johnson [42]	Fogelholm [43]
Case series collected in more than one centre, *i.e*., multi-centre study	0	0	0	0	0	0
Is the hypothesis/aim/objective of the study clearly described?	1	0	1	1	1	1
Are the inclusion and exclusion criteria (case definition) clearly reported?	1	1	1	0	1	1
Is there a clear definition of the outcomes reported?	0	1	1	1	1	1
Were data collected prospectively?	0	0	0	0	0	0
Is there an explicit statement that patients were recruited consecutively?	0	0	0	0	0	0
Are the main findings of the study clearly described?	1	1	1	0	1	1
Are outcomes stratified? (e.g., by disease stage, abnormal test results, patient characteristics)	0	1	0	0	1	1
Total Score	3	4	4	2	5	5

Yes = 1, No (not reported, not available) = 0; Total score, 8; ≤3, poor quality; 4–6, fair quality; ≥7, good quality; * Named by reference number and listed in chronological order.

### 4.1. Eating Disorders and Physical Fitness

Physical fitness is defined as a state of wellbeing with a low risk of premature health problems, and the energy to participate in a variety of physical activities, and in public health, it is one of the most important predictors for good health and the prevention of lifestyle-related diseases [49]. It is measured as the sum of morphological fitness, bone strength, muscular fitness, flexibility, motor fitness, cardiovascular fitness and metabolic fitness [50].

Table 1 summarizes the main findings of the study reviewed in this paper assessing physical fitness in eating disorders.

Several studies report a general reduction in muscular fitness in long-term eating disorder patients [20,22,26,31,51]. However, very little data is available on the various components of the physical fitness of such patients. Some studies observed lower muscle strength [23,26], and lower aerobic fitness [23,24,27,28,29], but no significant differences in postural control in comparison with healthy controls [30]. One study, comparing aerobic fitness (VO_2_max on treadmill) and muscle strength (1RM in leg and chest press) in 59 female in patients with long-standing eating disorder and 53 non-clinical controls, found a lower level of muscle strength in anorexia nervosa patients but no differences between the two groups in aerobic fitness [31].

The effects of refeeding and weight restoration on physical fitness in anorexia nervosa patients have also received some attention. Waller and colleagues reported a partial improvement in aerobic capacity over eight weeks of refeeding, but values were still lower than the norm [24]. Rigaud and colleagues, on the other hand, found that, despite low calf and thigh circumferences, muscular fitness (performance) was restored after 45 days of refeeding—long before normal weight regain [25]. Fohlin also reported the normalization of aerobic capacity after weight restoration in a small group of adolescents with anorexia nervosa [21]. These somewhat conflicting results may be the result of two main weaknesses in the methodology of these three studies. These were primarily that most patients assessed did not achieve complete weight restoration, and secondly, that only a few components of physical fitness were investigated.

Recently, Alberti and colleagues set out to overcome these two limitations by assessing 37 anorexia nervosa patients before and after complete weight restoration (≥18.5 kg/m^2^) [33]. Using a modified version of the Eurofit Physical Fitness Test Battery (EPFTB), a set of 6 physical fitness tests assessing aerobic fitness, musculoskeletal fitness*,* flexibility, and motor fitness [52], they found that patients with anorexia nervosa had significantly lower scores on all EPFTB tests than age-matched healthy controls. Five out of six EPFTB criteria improved significantly after weight restoration, but were still significant lower than the control group (all *p* < 0.001), except for muscular endurance. A possible explanation for this exception is that the muscular atrophy seen in anorexia nervosa patients, even if severe, tends to be type 2 [26], which, despite influencing maximal strength and explosive power, does not affect endurance [33]. As suggested by the authors of that study, the lack of total improvement in physical fitness after weight restoration may indicate that nutritional rehabilitation and weight regain are not sufficient to produce a complete restoration of physical fitness in patients with anorexia nervosa *per se*. Indeed, it may be that regaining normal fitness may require a long period of weight maintenance after weight restoration [33].

Some eating disorder treatment programmes combine nutritional rehabilitation with regular, systematic physical activity, but little has been published on the issue to date. Studies on muscular strength in treatment programmes featuring *light-to-moderate* intensity resistance training have thus far been inconclusive. One study found a positive effect of knee and elbow muscle strength after eight weeks of resistance training in anorexia nervosa patients [29], whereas another found no improvement in muscle strength after three months of resistance training [32].

### 4.2. Eating Disorders and Sport Performance

We found no study that examined the direct cause and effect relationship between eating disorders and sport performance, but some indirect or anecdotal (coach reports and accounts from athletes with eating disorders) information is available. There is also some data on the effect of specific eating disorder features (*i.e*., being underweight, excessive compulsive exercising, dietary restriction, binge eating and purging) on sport performance. Some of these features can be also observed in athletes who do not present the typical eating disorder psychopathology but rely on extreme weight control behaviour, “disordered eating”, to improve their sport performance [53]. Table 2 summarizes the main findings of the study assessing the effect of eating disorders features on sport performance reviewed in this paper.

#### 4.2.1. Underweight and Sport Performance

Very low body weight is a key clinical feature of anorexia nervosa, but can also be present in a subgroup of patients with eating disorder NOS [54]. Subgroups of not-underweight eating disorder patients with bulimia nervosa or eating disorder NOS may also present severe weight loss. Many athletes and coaches believe that weight loss and thinness can have a significant effect on sport performance [55,56], which may in part explain the higher prevalence of eating disorders in athletes. However, evidence to supporting this assumption is scarce. Studies conducted in the early 70 s on distance runners reported that leaner runners display better performance [34,35], but these data were not confirmed in studies conducted on female distance runners in the late 80 s and 90 s, which found no relationship between body weight and sport performance [36,38]. However, one study did report that medal-winning gymnasts tended to have lower body fat than the non-medal-winning gymnasts [57].

Another study found a correlation between low BMI and better performance among gymnasts participating in the world championships, but pointed out that this trend was reversed when BMI became very low [37]. Wilmore, commenting on this finding, suggested that although weight loss and low weight may enhance athletic performance in certain sports, there is a point beyond which continued weight loss produces a negative effect on performance, presumably due to the excessive loss of lean body mass and fluids [58], as seen in patients with anorexia nervosa [59]. In any case, when weight loss is severe, individuals with eating disorders develop so-called “starvation symptoms”, as described by Keys and colleagues in their classic “The Biology of Human Starvation” [60]. The onset of starvation symptoms, the most commonly reported by underweight eating disorder patients being preoccupation with thoughts about food and eating, irritability, mood changes, sleep disturbance, social withdrawal, weakness, gastrointestinal discomfort, cold intolerance, impaired concentration, apathy, and heightened satiety [61], will inevitably compromise sport performance.

#### 4.2.2. Excessive Compulsive Exercise and Sport Performance

Excessive and compulsive exercising is a common feature among eating disorder patients, particularly in those who are underweight [62,63]. Exercising is defined as “excessive” when it significantly interferes with important activities, occurs at inappropriate times or in inappropriate settings, or continues despite injury or other medical complications [64]. Exercising is defined as “compulsive” when it is associated with a subjective sense of being driven or compelled to exercise, giving it priority over other activities (e.g., school or work) and associating its postponement with feelings of guilt and anxiety [64].

The prevalence of excessive compulsive exercising among eating disorder patients ranges from 39% [63] to 45% [62], and can be as high as 80% in the restricting subtype of anorexia nervosa inpatients [62]. Indeed, excessive and compulsive exercising seems to play a significant role in the onset and maintenance of eating disorders [65], wherein it is usually used to control body weight and shape [66], but also to modulate mood [64]. It has been associated with increased risk of medical complications (e.g., overuse injuries, bone fractures, and cardiac complications) [67,68,69,70], higher rates of dropout from treatment programmes [71], longer inpatient treatment [72], poorer outcome [62], and quicker relapse [73]. It is also associated with greater severity of eating disorders [74] and general psychopathology, as well as specific personality features such as perfectionism and persistence [64]. It should be noted that many athletes may rely on their perfectionism, a trait associated with setting high goals and working hard to attain them [75], to help them succeed.

Most individuals with eating disorders practice excessive exercising in routine daily activities (e.g., walking for much of the day, standing rather than sitting while studying or watching television) or abnormal activities (e.g., doing extreme numbers of push-ups or sit-ups at home or in unusual places such as public restrooms), but some individuals adopt this behaviour in sports activities (e.g., training above and beyond a planned schedule or going to the gym several times a day) [64]. This form of exercising can be present both in competitive athletes and individuals who practice sports for recreation [76].

Unfortunately, excessive compulsive exercise is difficult to detect, especially in athletes, and usually only becomes apparent when they report fatigue, amenorrhea, weight loss, insomnia and/or deterioration in sport performance, a cluster of features known as “overtraining syndrome” [39]. This syndrome is a physical, behavioural, and emotional condition that manifests when the volume and intensity of an individual’s exercising exceeds their capacity for recovery [39]. In typical cases, athletes’ performance and progress begins to taper off, and they start losing strength and fitness. Overtraining is particularly likely to develop in athletes exposed to psychological stressors who engage in intense exercise while limiting their food intake [40], another cluster of factors common to individuals with eating disorders.

#### 4.2.3. Dietary Restriction and Sport Performance

As people with eating disorders judge themselves predominantly or exclusively in terms of their shape, weight and eating control, it is easy to understand why they usually adopt a strict dieting characterized by extreme, rigid dietary rules. Such rules govern when to eat (e.g., never before their evening meal), what to eat (e.g., only fruits and vegetables) and how much to eat (e.g., very small portions) [76]. Other typical rules include not eating with others or in the absence of hunger, eating less than others present, not eating food of uncertain calorie content or that prepared by others, and eating as late as possible. The persistent attempt to limit food intake (dietary restraint) may or may not produce a persistent calorie intake lower than a person’s energy expenditure and weight loss. In the first case the condition is defined as dietary restriction [76], and in the second case, typical of many individuals with bulimia nervosa or not-underweight eating disorder NOS, the attempt to reduce the calorie intake does not produce an energy deficit and weight loss because it is interrupted by recurrent bulimic episodes.

Extreme dietary restriction inevitably produces a deterioration in sport performance, but research into long-term undereating and sport performance is lacking, because confounding factors make it difficult to study this issue. Some coaches report that calorie restriction and initial weight loss may increase athletic performance, which Johnson [42] tentatively ascribed to the initial physiological and psychological excitability effects of starvation, mediated by up-regulation of the hypothalamic-pituitary-adrenal axis and an increase in cortisol, adrenaline and noradrenaline. Another proposed explanation is the initial increase of maximum oxygen intake (VO2max) associated with dietary restriction leading to an improvement in aerobic sport performance, at least in the short term [41,43]. However, in the long-term, the maintenance of dietary restriction produces a progressive deterioration of sport performance through several different mechanisms [43]. These include: (i) glycogen depletion causing a premature reduction in physical, psychological and mental capacity; (ii) circulatory lactate producing muscular pain; (iii) dehydration triggering muscular cramps; and (iv) loss of lean mass provoking a reduction in muscle strength and aerobic performance.

#### 4.2.4. Binge Eating and Purging and Sport Performance

Binge eating is a cardinal criterion for the diagnosis of bulimia nervosa and binge-eating disorder, but is also reported by individuals with anorexia nervosa of the binge-eating/purging type [44]. According to the DSM-5, an episode of binge eating is characterized both by eating an amount of food that would normally be regarded as too large, and by a sense of lack of control over eating [77]. These events are also described as objective bulimic episodes, to distinguish them from subjective bulimic episodes, in which the loss of control over eating is not associated with the consumption of what would generally be regarded as a large amount of food. Binge eating often occurs in individuals who adopt extreme and rigid dietary rules and tend to interpret any breaking of these as evidence of their lack of self-control. This prompts them to abandon their efforts to restrict their eating, resulting in a bulimic episode. This intensifies their concerns about their inability to control eating, shape and weight, and encourages further dietary restraint, which, in turn, increases the risk of further bingeing. Another process that maintains bulimic episodes is related to external events and associated mood changes. In this case, binge eating can be used to distract from concerns associated with negative life events and/or to mitigate intense, intolerable emotional states. Recurrent objective bulimic episodes, particularly in individuals with binge-eating disorder, may produce substantial weight gain [78], but no study has yet examined the acute health effects of overeating and weight gain in athletes [45].

Purging is another common, and very dangerous, behaviour adopted by some individuals with eating disorder [79]. The most common means of purging are self-induced vomiting, laxative misuse, diuretic misuse, and spitting with or without rumination (rare). In these individuals, purging has two main functions [76]: (i) to compensate for bulimic episodes (objective or subjective), *i.e*., compensatory purging; (ii) to reduce calorie intake irrespective of the amount of food ingested by taking, for example, large doses of laxatives or diuretics, *i.e*., non-compensatory purging.

Many athletes believe that purging can cause rapid and effective weight loss, preventing weight gain especially after binging, being ignorant of the fact that it is not an effective means of controlling weight. Self-induced vomiting is relatively ineffective at controlling weight because only part of the food ingested is expelled [80], while laxatives have very little effect on food absorption because they act in the large intestine, after the absorption of energy from nutrients has been completed in the small intestine. Diuretics have no effect on food absorption whatsoever, as they only eliminate liquid in the form of urine. However, both diuretics and laxatives have a short-lived effect on weight by causing dehydration (through loss of fluid in the form of urine or diarrhoea, respectively), but purging can easily foster bulimic episodes as individuals who believe in its effectiveness tend to relax their rigid control of food intake and subsequently binge eat.

Purging has a negative effect on sport performance through three main mechanisms [47]. In the first place it generates a negative caloric balance, in particular when self-induced vomiting is used after subjective bulimic episodes, provoking similar consequences on performance to that seen in diet-restricting and underweight individuals [43]. Secondly, as mentioned above, it causes dehydration through fluid depletion, leading to premature fatigue, increased risk of muscle cramps, and heatstroke [48]. Finally, especially in dehydrated individuals, it produces hypokalaemia, which is also associated with muscle fatigue and cramps [46].

## 5. Discussion

### 5.1. Summary of Evidence and Limitations

Few studies to date have assessed physical fitness and sport performance in individuals with eating disorders, and the information available in the literature is often indirect or theoretical in nature. However, all indications are that eating disorders have a detrimental effect on both physical fitness and sport performance. Long-term, eating disorder reduces the main qualities of muscular fitness (*i.e*., aerobic fitness, musculoskeletal fitness*,* flexibility, and motor fitness), which are not completely restored after nutritional rehabilitation and weight regain [33]. This finding is in line with the accounts of participants in the Human Starvation Study [60], who reported that the rehabilitation period was the most difficult part of the experiment, and that feelings of tiredness and weakness were slow to disappear [81]. These subjects also stated that they had not returned to normal activities by the end of the 3-month weight restoration period, and estimated that the time to achieve a full recovery ranged from 2 months to 2 years [81]. It is therefore plausible that for patients with eating disorders, a long period of weight maintenance after the weight restoration is required for them to return to normal fitness.

### 5.2. Implications for Future Research

Studies on successfully recovered eating disorder patients after a long period of healthy eating and weight maintenance will be needed to clarify the above hypothesis. Another interesting area for future research would be to investigate whether the inclusion of individualized health-enhancing physical activity regimes in eating disorder treatment programmes contributes to improving health-related physical fitness qualities [33]. As already reported, a healthy exercise regime seems to improve the quality of life and wellbeing of people with depression and serious mental illness by improving their physical health and alleviating psychiatric and social disability [82]. In addition, physical activity in the form of social exercising could help individuals with eating disorders to overcome social isolation, facilitate the acceptance of shape changes, give them practice in body exposure, and mitigate their urge to exercise excessively [83].

Nevertheless, to date we can only draw on studies and reports documenting the effects of particular clinical eating disorder features, such as low body weight, excessive compulsive exercising, dietary restriction, binge eating and purging, rather than specific eating disorders as a whole, on sport performance. Although each of these clinical features produces a deterioration in sport performance through different mechanisms, it is likely that their combination, common in individuals with eating disorder, has an even greater negative effect due to the presence of the eating disorder psychopathology itself (*i.e*., overvaluation of shape, weight and eating control) and the general psychiatric features (*i.e*., depression and anxiety) that often coexist. These are likely to have no small influence on athletes’ motivation to train and compete, and the resulting poor performance may tend to increase the pressure they feel to train harder and tighten their control of eating to lose more weight [84].

## 6. Conclusions

The information collated in this review has several practical implications. First, knowledge that significant improvements are associated with recovery could help to encourage both athletes and non-athletes with eating disorders to seek treatment, and to undertake the difficult task of regaining weight and addressing the other clinical features of their eating disorder. Second, athletics coaches should be a target for education about the risk factors for eating disorders, as they are potential “agents” for the early detection of eating disorders in their charges. They also should also be alerted to the fact that if an athlete shows reduced sport performance, in particular if they are underweight or display signs of dehydration, fatigue or amenorrhea, they may be suffering from an eating disorder, and should therefore be promptly referred to an eating disorder specialist for assessment and any necessary treatment. Both coaches and athletes alike should also be made aware that when athletes are underweight (*i.e*., BMI < 18.5) they should not be training or competing until they restore a healthy body weight [84]. Even if athletes with eating disorders are not underweight, they should only participate in training or competitions if they are in treatment, their sport participation is not symptomatic, they agree to increase their calorie intake to balance training, do not show marked signs of dehydration, and the treatment team determines that participation will not increase the athlete’s risk [84].
